# Supplementary material for: Predation and fragmentation portrayed in the statistical structure of prey time series
Source: BMC Ecol. 2009 May 6;9:10. doi: 10.1186/1472-6785-9-10 (PMC2689204; doi:10.1186/1472-6785-9-10)
Supplement: Additional file 2 — Voles and related classes ODDox Documentation. ODDox documentation of the agent-based model (ALMaSS) applied by Hendrichsen et al. The documentation is started by activating main.html. [file 1472-6785-9-10-S2.zip › Vole_ODDox/vole__all_8h.html]

ALMaSS ODDox: vole\_all.h File Reference

- Main Page
- Related Pages
- Classes
- Files

# vole\_all.h File Reference

---

## Detailed Description

**Vole\_all.h This header file contains the code for all vole lifestage classes**  

by Chris J. Topping   
Version of 01st Nov 2000   
All rights reserved.   
  
With additions as noted in:   
April 2006   
November 2007   
Doxygen formatted comments in May 2008   

|  |  |
| --- | --- |
|  |  |
| Classes | |
| class | struct\_Vole\_Adult |
|  | A struct for passing data to create a new vole. More... |
| class | Vole\_Base |
|  | Base class for voles - all vole objects are descended from this class. More... |
| class | Vole\_Female |
|  | The class for female voles. More... |
| class | Vole\_Male |
|  | The class for male voles. More... |
| Defines | |
| #define | \_\_SpecificPesticideEffectsVinclozolinLike\_\_ |
| Enumerations | |
| enum | TTypeOfVoleState {     tovs\_InitialState = 0, tovs\_JuvenileExploration, tovs\_MMaturation, tovs\_MEvaluateExplore,     tovs\_Infanticide, tovs\_MDying, tovs\_FEvaluateExplore, tovs\_ReproBehaviour,     tovs\_Lactating, tovs\_GiveBirth, tovs\_FMaturation, tovs\_Mating,     tovs\_UpdateGestation, tovs\_SpecialExplore, tovs\_FDying   } |
|  | Vole behavioural states. More... |

---

## Define Documentation

#define \_\_SpecificPesticideEffectsVinclozolinLike\_\_

---

## Enumeration Type Documentation

enum TTypeOfVoleState

Vole behavioural states.

**Enumerator:**
:   |  |  |
    | --- | --- |
    | *tovs\_InitialState* |  |
    | *tovs\_JuvenileExploration* |  |
    | *tovs\_MMaturation* |  |
    | *tovs\_MEvaluateExplore* |  |
    | *tovs\_Infanticide* |  |
    | *tovs\_MDying* |  |
    | *tovs\_FEvaluateExplore* |  |
    | *tovs\_ReproBehaviour* |  |
    | *tovs\_Lactating* |  |
    | *tovs\_GiveBirth* |  |
    | *tovs\_FMaturation* |  |
    | *tovs\_Mating* |  |
    | *tovs\_UpdateGestation* |  |
    | *tovs\_SpecialExplore* |  |
    | *tovs\_FDying* |  |

```
00037 {
00038       tovs_InitialState=0,
00039   //Males
00040       tovs_JuvenileExploration,
00041       tovs_MMaturation,
00042       tovs_MEvaluateExplore,
00043       tovs_Infanticide,
00044       tovs_MDying,
00045   //Females
00046       tovs_FEvaluateExplore,
00047       tovs_ReproBehaviour,
00048       tovs_Lactating,
00049       tovs_GiveBirth,
00050       tovs_FMaturation,
00051       tovs_Mating,
00052       tovs_UpdateGestation,
00053       tovs_SpecialExplore,
00054       tovs_FDying,
00055 } TTypeOfVoleState;
```

---

Generated on Thu Jan 22 14:13:45 2009 for ALMaSS ODDox by 
 1.5.6
